# Supplementary material for: Inflammatory factors and risk of lung adenocarcinoma: a Mendelian randomization study mediated by blood metabolites
Source: Front Endocrinol (Lausanne). 2024 Aug 27;15:1446863. doi: 10.3389/fendo.2024.1446863 (PMC11384989; doi:10.3389/fendo.2024.1446863)
Supplement: Supplementary file 1 [file DataSheet1.zip › supplementary files/S2/GCST90200391/sensitivity-analysis.pdf]

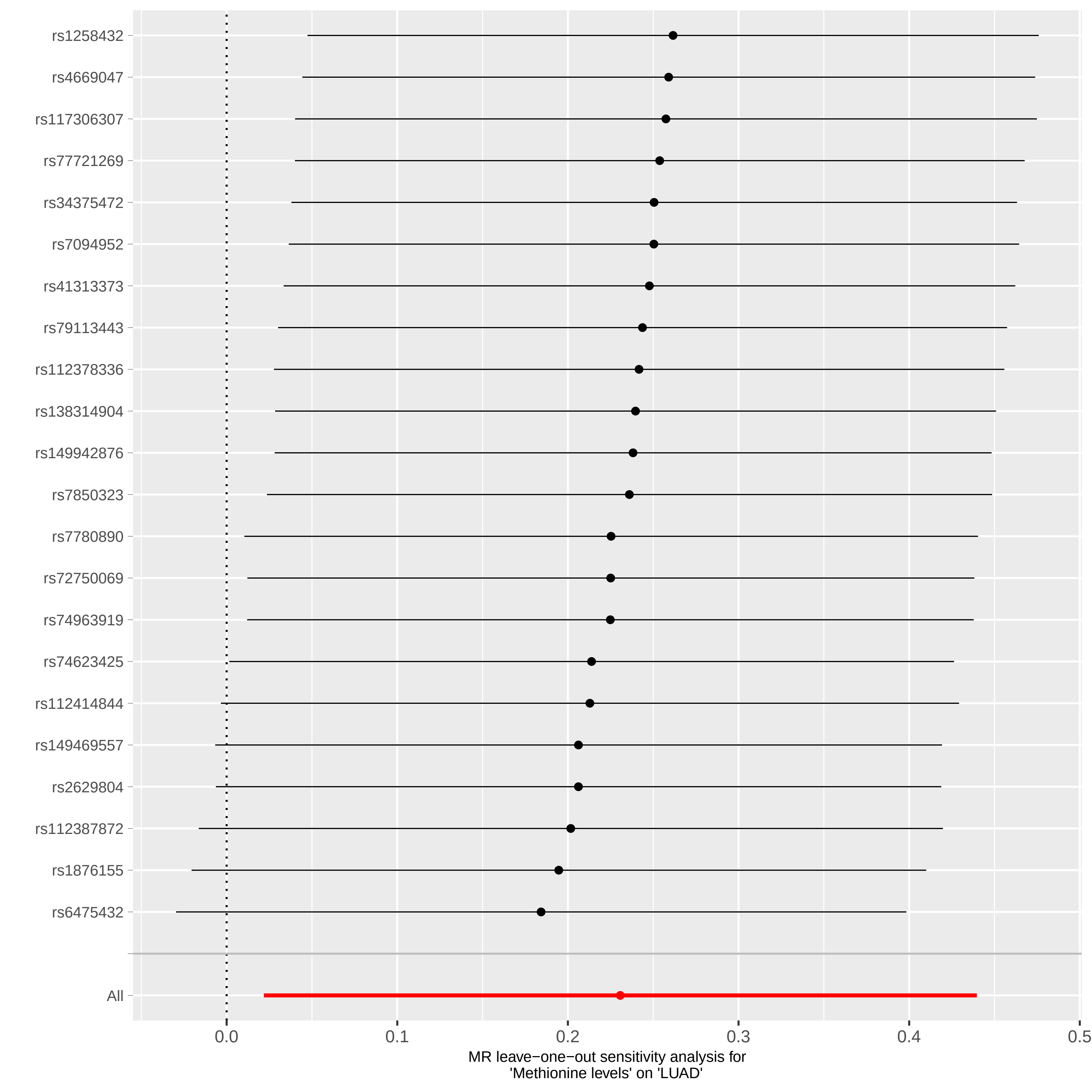

rs1258432
rs4669047
rs117306307
rs77721269
rs34375472
rs7094952
rs41313373
rs79113443
rs112378336
rs138314904
rs149942876
rs7850323
rs7780890
rs72750069
rs74963919
rs74623425
rs112414844
rs149469557
rs2629804
rs112387872
rs1876155
rs6475432
All
0.0
0.1
0.2
0.3
0.4
0.5
MR leave−one−out sensitivity analysis for
'Methionine levels' on 'LUAD'
